# Lifetime psychiatric hospital diagnoses among 8,412 Danish men registered in an outpatient alcohol clinic

**DOI:** 10.1002/brb3.2004

**Published:** 2021-01-09

**Authors:** Lea A. N. Christoffersen, Erik L. Mortensen, Merete Osler, Holger J. Sørensen, Ulrik Becker, Trine Flensborg‐Madsen

**Affiliations:** ^1^ Department of Public Health University of Copenhagen Copenhagen Denmark; ^2^ Center for Healthy Aging University of Copenhagen Copenhagen Denmark; ^3^ National Institute of Public Health University of Southern Denmark Copenhagen Denmark; ^4^ Center for Clinical Research and Prevention Bispebjerg and Frederiksberg Hospital Frederiksberg Denmark; ^5^ Mental Health Centre Copenhagen Copenhagen University Hospital Gentofte Hellerup Denmark

**Keywords:** Alcohol use disorder, Epidemiology, Mental disorders, RRID:SCR_008567, RRID:SCR_012763

## Abstract

**Objective:**

To describe the prevalence of lifetime psychiatric hospital diagnoses among men registered in an outpatient alcohol clinic and compare the prevalence with matched controls. To assess temporality of alcohol use disorder (AUD) diagnoses and another psychiatric hospital diagnosis and examine the prevalence of lifetime psychiatric hospital diagnoses according to this temporal order.

**Methods:**

The study included 8,412 Danish men registered in an outpatient alcohol clinic, and 8,412 unregistered controls from the Danish Conscription Database matched on birth date, lifespan, intelligence and draft board district. Information on first outpatient AUD treatment was retrieved from the Copenhagen Alcohol Cohort. Information on lifetime psychiatric hospital diagnoses was retrieved from national Danish psychiatric registers and based on the International Classification of Diseases the 8th and 10th Revisions. Prevalence estimates of lifetime psychiatric hospital diagnoses were compared with odds ratios (OR) between men registered in an outpatient alcohol clinic and the control population.

**Results:**

Among men registered in an outpatient alcohol clinic, 66.6% had a lifetime psychiatric hospital diagnosis. In total, 8.6% had neuroses and anxiety disorders, while 25.3% had personality disorders. The OR of a lifetime psychiatric hospital diagnosis was 9.77 (95%CI: 8.87–10.75) when comparing men registered in an outpatient alcohol clinic with the control population. Among men with a lifetime psychiatric hospital diagnosis, 42.8% was registered with another psychiatric hospital diagnosis before registration with an AUD diagnosis.

**Conclusion:**

Among men with a lifetime psychiatric hospital diagnosis, AUD is rarely diagnosed without psychiatric comorbidity at first‐time admissions to psychiatric hospital departments.

## Graphical abstract

Compared with a matched control population, lifetime psychiatric hospital diagnoses were prevalent in a population of men registered in an outpatient alcohol clinic. AUD is rarely diagnosed without psychiatric comorbidity at first‐time admissions to psychiatric hospital departments.

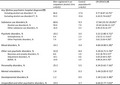

**Significant outcomes**

- In total, 59.4% of the men registered in an outpatient alcohol clinic had a lifetime AUD hospital diagnosis, indicating that studies based solely on psychiatric hospital diagnoses will underreport AUD and only include a selected group of individuals with psychiatric comorbidity.
- Compared with a matched control population, lifetime psychiatric hospital diagnoses are prevalent among men registered in an outpatient alcohol clinic.
- In total, 11.9% had an AUD hospital diagnosis prior to another psychiatric hospital diagnosis, indicating that AUD rarely is diagnosed without registration of co‐occurring psychiatric disorders at first‐time admissions to a psychiatric hospital department.

**Limitations**

- The prevalence of lifetime psychiatric hospital diagnoses may be underreported or lacking for some men because of the diagnostic practice used in the study. Moreover, psychiatric hospital diagnoses may be underreported for men who sought psychiatric treatment before 1969 where the national Danish psychiatric registers were established, or for men who sought treatment in other settings than a psychiatric hospital department.
- Psychiatric hospital diagnoses were based on the International Classification of Diseases the 8th and 10th Revisions, possibly implying that different phenotypes have been identified.
- The probability of being registered with a lifetime psychiatric hospital diagnosis may be different in the study sample compared with the general population or individuals receiving other types of AUD treatment; thus, the results cannot be generalized to all individuals with AUD.

## 1 INTRODUCTION

Individuals with alcohol use disorders (AUD) have a high prevalence of psychiatric comorbidity. This has been demonstrated in population surveys assessing the 12‐month (Farrell et al., 2003; Grant et al., 2004) and lifetime (Cohen et al., 2007; Goldstein et al., 2012) prevalence of psychiatric comorbidity among individuals fulfilling the diagnostic criteria for AUD (Cohen et al., 2007; Farrell et al., 2003; Goldstein et al., 2012; Grant et al., 2004; Lai et al., 2015). For example, one population survey found that almost one‐third of individuals fulfilling the diagnostic criteria for AUD had psychiatric comorbidity (Farrell et al., 2003). In some of these studies, personality disorders (Goldstein et al., 2012) and other substance use disorders (Cohen et al., 2007; Goldstein et al., 2012) were the most prevalent co‐occurring disorders among individuals fulfilling the diagnostic criteria for AUD. In addition, mood disorders and anxiety disorders have been observed to be relatively prevalent among individuals fulfilling the diagnostic criteria for AUD (Cohen et al., 2007; Dawson et al., 2005; Goldstein et al., 2012; Grant et al., 2004, 2015; Lai et al., 2015).

The results from the population surveys are similar to results on psychiatric comorbidity from studies of individuals receiving AUD treatment, assessing both the lifetime prevalence (Flensborg‐Madsen et al., 2009; Gale et al., 2010; Mortensen et al., 2005; Tomasson & Vaglum, 1995) and the 4‐ to 6‐ month prevalence (Kushner et al., 2005; Schneider et al., 2001) of co‐occurring disorders. Thus, two studies observed that at least 50% of individuals receiving AUD treatment had psychiatric comorbidity (Flensborg‐Madsen et al., 2009; Tomasson & Vaglum, 1995). In addition, studies that included personality disorders found these disorders to be the most prevalent among individuals receiving AUD treatment (Flensborg‐Madsen et al., 2009; Mortensen et al., 2005). Moreover, numerous studies reported that substance use disorders were prevalent among individuals receiving AUD treatment (Flensborg‐Madsen et al., 2009; Gale et al., 2010; Mortensen et al., 2005; Tomasson & Vaglum, 1995). Several studies of individuals receiving AUD treatment also included mood disorders (Flensborg‐Madsen et al., 2009; Gale et al., 2010; Schneider et al., 2001; Tomasson & Vaglum, 1995) and anxiety disorders (Gale et al., 2010; Kushner et al., 2005; Schneider et al., 2001; Tomasson & Vaglum, 1995) and found that both types of disorders were relatively prevalent among individuals receiving AUD treatment. In contrast, none of the studies reported a large prevalence of psychotic disorders among individuals receiving AUD treatment (Flensborg‐Madsen et al., 2009; Gale et al., 2010; Mortensen et al., 2005; Tomasson & Vaglum, 1995).

The population surveys and the studies of individuals receiving AUD treatment included various psychiatric disorders. However, only one of these studies included cognitive impairment (Tomasson & Vaglum, 1995), while none of them included developmental disorders as a separate category (Cohen et al., 2007; Dawson et al., 2005; Farrell et al., 2003; Flensborg‐Madsen et al., 2009; Gale et al., 2010; Goldstein et al., 2012; Grant et al., 2004, 2015; Kushner et al., 2005; Lai et al., 2015; Mortensen et al., 2005; Schneider et al., 2001; Tomasson & Vaglum, 1995), limiting conclusions about mental retardation and developmental disorders among individuals with AUD. In addition, some of the studies only reported the prevalence of the two or three most common co‐occurring disorders among individuals with AUD (Gale et al., 2010; Mortensen et al., 2005), leaving the prevalence of other co‐occurring disorders unassessed. Consequently, studies including a broad range of psychiatric disorders are needed to obtain a full description of psychiatric comorbidity among individuals with AUD. Finally, the previous studies were based on general population samples assessing a wide range of co‐occurring disorders (Cohen et al., 2007; Dawson et al., 2005; Farrell et al., 2003; Goldstein et al., 2012; Grant et al., 2004, 2015; Lai et al., 2015) or individuals primarily receiving inpatient psychiatric treatment (Flensborg‐Madsen et al., 2009; Gale et al., 2010; Kushner et al., 2005; Mortensen et al., 2005; Schneider et al., 2001; Tomasson & Vaglum, 1995). Therefore, these studies may to a large extent reflect how psychiatric disorders co‐occur rather than showing the prevalence of psychiatric comorbidity among individuals with a specific psychiatric disorder such as AUD. This is also indicated by the large prevalence of AUD observed among individuals with other psychiatric disorders in some of the previous studies (Gale et al., 2010; Mortensen et al., 2005). Addressing this issue can be difficult, but one possible solution is to study psychiatric comorbidity among individuals who were exclusively treated for AUD, for example, in outpatient alcohol clinics. However, studies of individuals receiving outpatient AUD treatment have mostly focused on personality disorders, mood disorders, and anxiety disorders and the results were relatively heterogeneous as both current prevalence estimates (Mellentin et al., 2015, 2018; Nordholm & Nielsen, 2007; Smith & Book, 2010; Yoshimi et al., 2016; Zikos et al., 2010), 12‐month prevlences estimates (Burns et al., 2005), and lifetime prevalence estimates (Nordholm & Nielsen, 2007) were reported. Thus, the prevalence of personality disorders has been observed to range between 34% and 59% (Nordholm & Nielsen, 2007; Zikos et al., 2010), while the prevalence of mood disorders ranged from 15.7% to 58.0% (Burns et al., 2005; Mellentin et al., 2015; Nordholm & Nielsen, 2007). Finally, the prevalence of anxiety disorders has been observed to range from 12.7% to 46.2% (Burns et al., 2005; Mellentin et al., 2015, 2018; Nordholm & Nielsen, 2007; Smith & Book, 2010; Yoshimi et al., 2016).

In addition to the results on the prevalence of psychiatric comorbidity among individuals with AUD, some population surveys also compared the prevalence of psychiatric comorbidity among individuals with AUD and individuals without AUD. One study found that the 12‐month prevalence of psychiatric comorbidity among individuals with AUD was more than double the prevalence among individuals without AUD (Farrell et al., 2003). Moreover, among individuals receiving AUD treatment the rates of any mood disorder and any anxiety disorder were, respectively, around 2.5 times and 3 times larger when compared with individuals without AUD (Schuckit et al., 1997). In addition, results from population surveys have shown that individuals with AUD had significantly higher odds ratios of lifetime personality disorders (Hasin et al., 2007), substance use disorders (Hasin et al., 2007; Ross, 1995), mood disorders (Hasin et al., 2007; Ross, 1995), and anxiety disorders (Hasin et al., 2007; Ross, 1995) compared with individuals without AUD. However, none of these studies compared the prevalence of psychiatric comorbidity between individuals receiving AUD treatment (without including other substance use disorders) and a matched control population, making it difficult to evaluate the prevalence of psychiatric comorbidity among individuals receiving AUD treatment.

When examining psychiatric comorbidity among individuals with AUD, it may be interesting to assess the prevalence estimates according to the temporal order of registration with AUD and registration of another psychiatric disorder, because it may reflect treatment‐seeking patterns and diagnostic traditions. However, to our knowledge, previous studies have not included such an assessment.

### 1.1 Aims of the study

First, to describe the lifetime prevalence of psychiatric hospital diagnoses among 8,412 men registered in an outpatient alcohol clinic. Second, to compare the lifetime prevalence of psychiatric hospital diagnoses between the 8,412 men registered in an outpatient alcohol clinic and a matched control population of 8,412 men never registered in an outpatient alcohol clinic. Third, to examine the temporal order of registration with an AUD diagnosis and registration with another psychiatric hospital diagnosis and to describe the lifetime prevalence of psychiatric hospital diagnoses among men registered in an outpatient alcohol clinic according to this temporal order.

## 2 MATERIALS AND METHODS

### 2.1 Ethical considerations

According to Danish law, register‐based studies should not be evaluated by the Scientific Ethics Committee system and do not require informed consent from participants.

### 2.2 Study population

This register‐based cohort study was based on a sample of men registered in the Copenhagen Alcohol Cohort that contains information on 32,098 Danish men and women who received AUD treatment in five outpatient clinics between 1954 and 2009 in Copenhagen, Denmark (Holst et al., 2017; Thygesen et al., 2009).

#### 2.2.1 The control population

The study also included a matched control population from the Danish Conscription Database (DCD), containing information on 728,160 Danish men born between 1939 and 1959 (Christensen et al., 2015). A person was defined as a case upon first registration in an outpatient alcohol clinic, while controls were defined as never registered in an outpatient alcohol clinic. Hence, men from the control population could not be included as cases and vice versa. In the present study, the purpose of including a control population was to assess the lifetime prevalence of psychiatric hospital diagnoses among men registered in an outpatient alcohol clinic while accounting for factors potentially associated with the development of psychiatric disorders including date of birth, lifespan, intelligence, and draft board district. Hence, the control population was selected to reflect the study sample of men registered in an outpatient alcohol clinic on the matching parameters and was not necessarily representative of the Danish background population.

#### 2.2.2 Subpopulations

Men registered in an outpatient alcohol clinic who had a lifetime psychiatric hospital diagnosis were divided into three subpopulations according to the temporality of registration with an AUD diagnosis and registration with another psychiatric hospital diagnosis: *The AUD first subpopulation* included men registered in an outpatient alcohol clinic prior to registration with a psychiatric hospital diagnosis or men registered with an AUD hospital diagnosis prior to registration with another psychiatric hospital diagnosis; *the other diagnosis first subpopulation* contained men registered with another psychiatric hospital diagnosis than AUD prior to registration in an outpatient alcohol clinic or registration with an AUD hospital diagnosis; *the simultaneous diagnosis subpopulation* included men who were registered with an AUD diagnosis and another psychiatric hospital diagnosis at the same age.

### 2.3 Exclusion criteria

**Figure 1:**
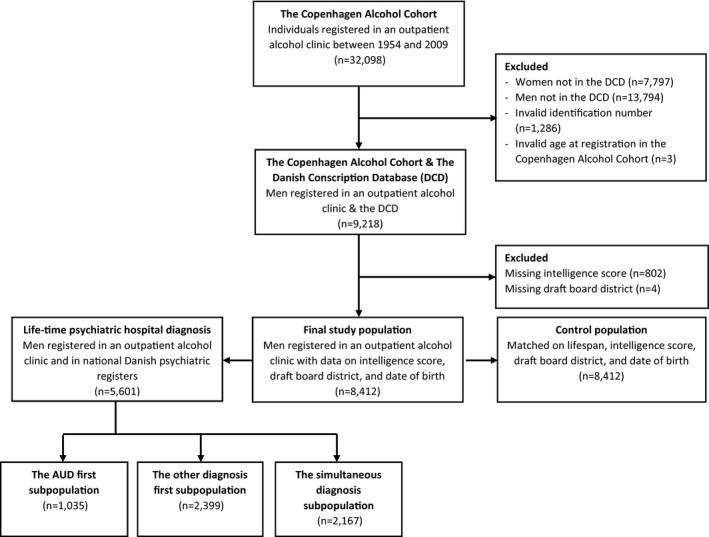
The study population

As the control population was drawn from the DCD, the study population had to be registered in this database. Accordingly, all women (*n* = 7,797) were excluded from the study as the DCD only contains men, while 13,794 men were excluded because their birth cohorts were not included in the DCD. The unique personal identification number assigned to all Danish residents was used to link information between different registers. Therefore, 1,286 men (primarily non‐Danish residents) were excluded because they had an invalid identification number. Next, 3 men were excluded because their age at first registration in an outpatient alcohol clinic was invalid. Finally, men with missing information on intelligence score (*n* = 802), and draft board district (*n* = 4) were excluded (Figure 1). Thus, the present study included 8,412 men registered in one of the five outpatient alcohol clinics between 1959 and 2009 and a matched control population of 8,412 unregistered men.

### 2.4 Lifetime psychiatric hospital diagnoses

The lifetime psychiatric disorders included in the present study were disorders exclusively diagnosed in psychiatric hospital departments (i.e., psychiatric hospital diagnoses) in Denmark between 1 April 1969 and 22 March 2016. In these departments, psychiatrists have the primary responsibility for the diagnoses, which are based on contact with the patient during admission or outpatient contacts. During admission to a psychiatric hospital department, the main diagnosis and, if relevant, several secondary diagnoses are reported to the national Danish psychiatric registers (the Danish Psychiatric Central Research Register and the Danish National Patient Registry) that have existed since 1969. In Denmark, very few private psychiatric hospitals exist. Hence, the national Danish psychiatric registers have a high coverage rate of 95%–100% of all admissions to psychiatric hospital departments in Denmark (Lynge et al., 2011; Munk‐Jorgensen & Mortensen, 1997). From 1969 to 1974, the registers included inpatient admissions only. Psychiatric day admissions have been registered since 1974, while outpatient admissions and emergency admissions have been recorded since 1995 (Lynge et al., 2011; Munk‐Jorgensen & Mortensen, 1997).

The psychiatric hospital diagnoses in the present study were based on the International Classification of Diseases (ICD) including both the 8th Revision (ICD‐8) and the 10th Revision (ICD‐10). The two diagnostic systems rely on different diagnostic approaches, potentially leading to different classification of phenotypes. To assess possible differences between ICD‐8 and ICD‐10, a sensitivity analysis was conducted, examining the prevalence of each psychiatric disorder according to ICD‐8 and ICD‐10, respectively. Generally, no marked differences were observed, but some disorders (e.g., developmental disorders) were more often diagnosed by ICD‐8, while other disorders (e.g., mood disorders) were more often diagnosed by ICD‐10. As ICD‐8 was only used until 1994 in Denmark, these differences may largely reflect that some disorders are more likely to be diagnosed early in life, while other disorders often are diagnosed later.

Table 1 lists the lifetime psychiatric hospital diagnoses that were included in the study according to ICD‐8 and ICD‐10, respectively. The disorders were divided into the following categories: *substance use disorders* (alcohol use disorders and other substance use disorders); *psychotic disorders* (schizophrenia and other psychotic disorders); *mood disorders*; *other non‐psychotic disorders* (neuroses and anxiety disorders, adjustment disorders, and behavioral syndromes associated with physiological disturbances and physical factors [BSPDF]); *personality disorders*; *mental retardation; developmental disorders*; and *unspecified and other psychiatric disorders*.

**Table 1:** Lifetime psychiatric hospital diagnoses included in the study according to ICD8‐codes and ICD10‐codes

	ICD8‐codes	ICD10‐codes
*Substance use disorders*		
Alcohol use disorders^a^	303;291; 980.3; 980.9	F10
Other substance use disorders	304	F11‐F19
*Psychotic disorders*		
Schizophrenia	295	F20‐F21
Other psychotic disorders	297–298	F22‐F29
*Mood disorders*	296	F30‐F39
*Other non‐psychotic disorders*		
Neuroses and anxiety disorders	300; 305	F40‐F42; F44‐F49
Adjustment disorders	307	F43
BSPDF	290;292–294; 309	F00‐F09; F50‐F59
*Personality disorders*	301–302	F60‐F69
*Mental retardation*	310–315	F70‐F79
*Developmental disorders*	299;306.0–306.5;306.7;306.9; 308.0;308.3–308.4;	F80‐F98
*Unspecified and other psychiatric disorders*	793;980; unspecified	F99; unspecified

Abbreviations: BSPDF, behavioral syndromes associated with physiological disturbances and physical factors; ICD8, International Classification of Diseases 8th Revision; ICD10, International Classification of Diseases 10th Revision

^a^ In national Danish psychiatric registers

### 2.5 Statistical analyses

Statistical analyses were performed in SAS version 9.4 (Statistical Analysis System, RRID:SCR_008567) and Stata (Stata, RRID:SCR_012763) version 16 with a significance level of 5%. All analyses were explorative with no critical hypothesis, and therefore, the results were not corrected for multiple statistical tests.

#### 2.5.1 Matching procedure

The study sample and the control population were matched on date of birth, lifespan, intelligence, and draft board district. Draft board district was matched on a one‐to‐one basis, while nearest neighbor propensity score matching was used for the other three variables (sampling the controls without replacement). The propensity score was estimated by the use of logistic regression as the relative odds of receiving AUD treatment conditional on date of birth, lifespan, and intelligence.

#### 2.5.2 Matching variables

##### Date of birth

Date of birth was retrieved from the DCD and used as a matching criterion because the level of intelligence (Flynn, 1984, 1987; Teasdale & Owen, 1987) and registration of psychiatric hospital diagnoses can vary over time. Date of birth was compared between the study sample and the control population with a paired *t* test. After the matching, a mean difference in date of birth of 0.23 years existed (*t*(8,411)=−2.91, *p* = .0037) between the study sample and the control population.

##### Lifespan

Lifespan was defined as the time from birth until date of death or end of follow‐up in the national Danish psychiatric registers (22. March 2016). Information on the date of death was retrieved from the Danish Register of Causes of Death that holds information on all deaths in Denmark since 1970, where the register was computerized (Helweg‐Larsen, 2011). Lifespan was used as a matching criterion because the exposure time (i.e., the time where an individual can receive a lifetime psychiatric hospital diagnosis) must be similar in the study sample and the control population. A paired *t* test was used to compare the lifespan between the study sample and the control population. After the matching, the mean lifespan was 58.69 (*SD* = 10.4) years in the study sample and 59.00 (*SD* = 11.6) years in the control population (t(8,411)=−3.30, *p* = .001).

##### Intelligence

Intelligence was obtained from the DCD and used as a matching criterion because low intelligence has been associated with a higher risk of developing AUD (Sjolund et al., 2012, 2015). In addition, intelligence has been observed to differ significantly between individuals with and without a psychiatric disorder (Mortensen et al., 2005) and between individuals with different psychiatric disorders (Urfer‐Parnas et al., 2010). In the present study, intelligence was measured with the intelligence test Børge Prien's Prøve (BPP). The total test score of BPP ranges from 0 to 78 (Teasdale, 2009), and the mean BPP score among the men in the DCD is 37.8 (*SD* = 12.0) (Christensen et al., 2015). BPP is strongly correlated with the full Wechsler Adult Intelligence Scale (*r* = 0.82), indicating that BPP is a high‐quality measure of global intelligence (Mortensen et al., 1989). In addition, BPP has been observed to have a high test–retest coefficient of 0.81 when the test was administered with a mean test–retest interval of 41 years (Grønkjær et al., 2019). The mean intelligence score was compared between the study sample and the control population with a paired *t* test. After the matching, the mean intelligence score in the study sample was 34.72 (*SD* = 12.5), while the mean intelligence score in the control population was 33.86 (*SD* = 12.2) *t*(8,411)= 6.00, *p* < .001).

##### Draft board district

In the DCD, Denmark is divided into seven geographical draft board districts, covering the entire country (Christensen et al., 2015). The study sample and the control population was matched on a one‐to‐one basis on this parameter because the area of residence affects the probability of being registered in an outpatient alcohol clinic, as the clinics were only located in Copenhagen.

#### 2.5.3 Comparisons with the control population

The lifetime prevalence of each psychiatric hospital diagnosis was assessed by percentages among the 8,412 men registered in an outpatient alcohol clinic and in the matched control population of 8,412 men never registered in an outpatient alcohol clinic. To compare the lifetime prevalence of psychiatric hospital diagnoses between men registered in an outpatient alcohol clinic and the control population, odds ratios (OR) for dependent groups were calculated in a logistic regression model. The model contained an indicator variable describing if the individuals belonged to the study sample or the control population in addition to the matching variables that differed significantly between the groups (date of birth, lifespan, and intelligence). The model calculated a Wald chi‐square test with 1 degree of freedom.

#### 2.5.4 Temporality of registration with an AUD diagnosis and another psychiatric hospital diagnosis

To assess the temporality of registration with an AUD diagnosis and registration with another psychiatric hospital diagnosis, age at first AUD diagnosis and age at first psychiatric hospital diagnosis were calculated. Age at first AUD diagnosis was defined as age at first outpatient alcohol treatment or age at first AUD hospital diagnosis, using whichever occurred at the youngest age. Age at first outpatient AUD treatment was retrieved from the Copenhagen Alcohol Cohort. The date of the first AUD hospital diagnosis was retrieved from the national Danish psychiatric registers and subtracted from the date of birth to reach the age at the first AUD hospital diagnosis. Age at first psychiatric hospital diagnosis was calculated by subtracting the date of first registration with a psychiatric hospital diagnosis from the date of birth.

The lifetime prevalence of each psychiatric hospital diagnosis was compared pairwise between the AUD first subpopulation, the other diagnosis subpopulation, and the simultaneous diagnosis subpopulation with a chi‐square test for equal proportions, and overall p‐values were examined with chi‐square tests.

## 3 RESULTS

### 3.1 Lifetime psychiatric hospital diagnoses among men registered in an outpatient alcohol clinic

Of the 8,412 men registered in an outpatient alcohol clinic, 66.6% (*n* = 5,601) had been admitted to a psychiatric hospital department and had at least one lifetime psychiatric hospital diagnosis including AUD (Table 2). The prevalence of lifetime psychiatric hospital diagnoses without including AUD was 53.3%.

**Table 2:** Lifetime psychiatric hospital diagnoses among 8,412 Danish men registered in an outpatient alcohol clinic and a matched control population of 8,412 Danish men never registered in an outpatient alcohol clinic

	Men registered in an outpatient alcohol clinic *n* = 8,412	Control population^d^ *n* = 8,412	OR (95% CI)^e^, ^f^
*Any lifetime psychiatric hospital diagnosis* ^g^			
Including alcohol use disorders^a^, %	66.6	17.6	9.77 (8.87–10.75)*
Excluding alcohol use disorders^b^, %	53.3	15.6	6.26 (5.74–6.82)*
*Substance use disorders,%*	60.6	9.3	17.64 (15.52–20.05)*
Alcohol use disorders^c^, %	59.4	7.9	19.34 (16.94–22.12)*
Other substance use disorders, %	16.4	3.3	6.95 (5.91–8.17)*
*Psychotic disorders, %*	10.2	3.5	3.22 (2.80–3.71)*
Schizophrenia, %	5.4	2.3	2.52 (2.11–3.01)*
Other Psychotic disorders, %	7.4	2.3	3.61 (3.03–4.29)*
*Mood disorders, %*	14.1	3.4	4.60 (4.00–5.28)*
*Other non‐psychotic disorders, %*	32.0	8.4	5.20 (4.71–5.74)*
Neuroses and anxiety disorders, %	8.6	2.0	4.51 (8.79–5.37)*
Adjustment disorders, %	19.1	4.6	5.03 (4.44–5.70)*
BSPDF, %	14.9	3.4	4.99 (4.34–5.74)*
*Personality disorders, %*	25.3	5.5	6.34 (5.62–7.16)*
*Mental retardation, %*	1.4	0.3	5.44 (3.05–9.72)*
*Developmental disorders,%*	1.2	0.5	2.34 (1.62–3.38)*
*Unspecified and other psychiatric disorders, %*	19.5	3.7	6.64 (5.77–7.63)*

Abbreviation: BSPDF, behavioral syndromes associated with physiological disturbances and physical factors.

^a^ Including alcohol use disorder hospital diagnosis

^b^ Excluding alcohol use disorder hospital diagnoses

^c^ In national Danish psychiatric registers

^d^ Never registered in an outpatient alcohol clinic, matched on date of birth, intelligence, lifespan, and draft board district,

^e^ The control population was the reference group

^f^ Adjusted for the differences on the matching variables

^g^ The mean lifespan among men with a psychiatric hospital diagnosis was 58.46 years, while the mean lifespan among men without a psychiatric hospital diagnosis was 58.94 years (*p* = .0034).

* *p* < .001

*Substance use disorders* were the most prevalent category of disorders, and as expected, AUD was the most prevalent lifetime psychiatric hospital diagnosis with a prevalence of 59.4%. With a prevalence estimate of 32.0%, *other non‐psychotic disorders* were also prevalent among men registered in an outpatient alcohol clinic and this category included neuroses and anxiety disorders (8.6%), adjustment disorders (19.1%), and BSPDF (14.9%). In addition, *personality disorders* were prevalent with a prevalence estimate of 25.3%. Finally, the prevalence of *mental retardation* (1.4%) and *developmental disorders* (1.2%) were low among men registered in an outpatient alcohol clinic.

### 3.2 Comparisons with the control population

The OR of having a lifetime psychiatric hospital diagnosis was more than nine times larger (OR = 9.77 [95% CI: 8.87–10.75]) among men registered in an outpatient alcohol clinic compared with the control population (Table 2). In fact, men registered in an outpatient alcohol clinic had significantly higher ORs of all lifetime psychiatric hospital diagnoses compared with the control population. The OR of AUD was 19.34 (95% CI: 16.94–22.12) when comparing men registered in an outpatient alcohol clinic with the control population. In addition, the ORs of *substance use disorders* (OR = 17.64 [95% CI:15.52–20.05]), *mood disorder* (OR = 4.60 [95% CI:4.00–5.28]), *other non‐psychotic disorders* (OR = 5.20 [95% CI: 4.71–5.74]), *personality disorders* (OR = 6.34 [95% CI: 5.62–7.16]), and *mental retardation* (OR = 5.44 [95% CI: 3.05–9.72]) were relatively high when comparing men registered in an outpatient alcohol clinic with the control population. The ORs for *psychotic disorders* (OR = 3.22 [95% CI: 2.80–3.71]) and *developmental disorders* (OR = 2.34 [95% CI: 1.62–3.38]) were lower, but significantly higher among men registered in an outpatient alcohol clinic compared with the control population.

### 3.3 Temporal order of registration with an AUD diagnosis and another psychiatric hospital diagnosis

Table 3 shows the temporal order of registration with an AUD diagnosis and registration with another psychiatric hospital diagnosis among men registered in an outpatient alcohol clinic. Among men with a lifetime psychiatric hospital diagnosis, 1,035 (18.5%) were registered with an AUD diagnosis prior to another psychiatric hospital diagnosis (*the AUD first subpopulation*). Of the 1,035 men in the AUD first subpopulation, 371 (35.8%) had their first AUD diagnosis in an outpatient alcohol clinic, 485 (46.9%) had their first AUD diagnosis in a psychiatric hospital department, while 179 (17.3%) had an AUD diagnosis from an outpatient alcohol clinic and psychiatric hospital department at the same age. In total, 2,399 (42.8%) were registered with another psychiatric hospital diagnosis prior to an AUD diagnosis (*the other diagnosis first subpopulation*), while 2,167 (38.7%) were registered with an AUD diagnosis and another psychiatric hospital diagnosis at the same age (*the simultaneous diagnosis subpopulation*).

**Table 3:** Lifetime psychiatric hospital diagnoses among 5,601 Danish men registered in an outpatient alcohol clinic according to the temporality of registration with an AUD diagnosis and another psychiatric hospital diagnosis

	1. The AUD first subpopulation *n* = 1,035 (18.5%)	2. The other diagnosis first subpopulation *n* = 2,399 (42.8%)	3. The simultaneous diagnosis subpopulation *n* = 2,167 (38.7%)	Overall ꭓ^2^ (*df*)
*Substance use disorders, %*	74.5^2,3^	92.0^1,3^	98.0^1,2^	478.2 (2)**
Alcohol use disorders^a^, %	70.9^2,3^	89.5^1,3^	97.5^1,2^	513.8 (2)**
Other substance use disorders, %	19.1^3^	20.1^3^	32.2^1,2^	109.9 (2)**
*Psychotic disorders, %*	12.1^3^	13.6^3^	18.9^1,2^	35.8 (2)**
Schizophrenia, %	6.7^3^	7.8^3^	9.3^1,2^	7.4 (2)*
Other psychotic disorders, %	8.2^2,3^	10.0^1,3^	13.8^1,2^	28.0 (2)**
*Mood disorders, %*	26.9^2,3^	15.8^1,3^	24.2^1,2^	72.8 (2)**
*Other non‐psychotic disorders, %*	52.2^2,3^	35.6^1,3^	59.8^1,2^	276.1 (2)**
Neuroses and anxiety disorders, %	12.7^3^	11.0^3^	15.2^1,2^	18.5 (2)**
Adjustment disorders, %	25.8^2,3^	22.7^1,3^	36.7^1,2^	115.2 (2)**
BSPDF, %	23.5^2,3^	15.8^1,3^	29.2^1,2^	117.1 (2)**
*Personality disorders, %*	24.5^2,3^	28.5^1,3^	54.8^1,2^	1706.4 (2)**
*Mental retardation, %*	1.4^3^	1.4^3^	3.3^1,2^	24.4 (2)**
*Developmental disorders, %*	1.3	1.9	1.9	2.1 (2)
*Unspecified and other psychiatric disorders, %*	39.7^2,3^	22.5^1^	32.0^1,2^	115.9 (2)**

Numbered superscripts indicate subpopulations that differ significantly at *p* ≤ .05

Abbreviations: BSPDF, behavioral syndromes associated with physiological disturbances and physical factors; *df*, degrees of freedom

^a^ In national Danish psychiatric registers.

** *p* < .001

* *p* < .02

*Substance use disorders* were prevalent in all three subpopulations, but the simultaneous diagnosis subpopulation had a significantly higher prevalence of substance use disorders (98.0%) compared with the other two subpopulations, while the AUD first subpopulation had a significantly lower prevalence of substance use disorders (74.5%) when comparing with the other two subpopulations. The same pattern was observed for AUD (Table 3).

Almost all psychiatric hospital diagnoses including *psychotic disorders* (18.9%), *other non‐psychotic disorders* (59.8%), *personality disorders* (54.8%), and *mental retardation* (3.3%) were significantly more prevalent in the simultaneous diagnosis subpopulation compared with the other two subpopulations. Only *mood disorders* followed a different pattern and were more prevalent in the AUD first subpopulation (26.9%) compared with the other two subpopulations. In contrast, the prevalence of other psychotic disorders than schizophrenia (8.2%) and *personality disorders* (24.5%) was significantly lower in the AUD first subpopulation when comparing with the other two subpopulations. Finally, the prevalence of both *mood disorders* (15.8%) and *other non‐psychotic disorders* (35.6%) was significantly lower in the other diagnosis first subpopulation when comparing with the other two subpopulations.

## 4 DISCUSSION

In this description of lifetime psychiatric hospital diagnoses among 8,412 Danish men registered in an outpatient alcohol clinic, approximately two‐thirds (66.6%) had been registered in a psychiatric hospital department and received a lifetime psychiatric hospital diagnosis. Not surprisingly, AUD was the most prevalent lifetime psychiatric hospital diagnosis among men registered in an outpatient alcohol clinic, but *other non‐psychotic disorders,* including neuroses and anxiety disorders, adjustment disorders, and behavioral syndromes associated with physiological disturbances and physical factors, and *personality disorders* were also prevalent. The OR of having a lifetime psychiatric hospital diagnosis was more than nine times higher among men registered in an outpatient alcohol clinic compared with the control population. The majority of men with a lifetime psychiatric hospital diagnosis was registered with another psychiatric hospital diagnosis prior to registration with an AUD diagnosis.

### 4.1 Lifetime psychiatric hospital diagnoses among men registered in an outpatient alcohol clinic

In the present study, *substance use disorders* were the most frequent category of psychiatric hospital diagnoses with a prevalence estimate of 60.6%. This is not surprising as the study was based on men registered in an outpatient alcohol clinic, which may be expected to result in a high number of AUD hospital diagnoses. In total, 59.4% of the men registered in an outpatient alcohol clinic had an AUD hospital diagnosis, indicating that far from all men at some point had been registered in the national Danish psychiatric registers with an AUD hospital diagnosis. Consequently, the national Danish psychiatric registers will substantially underreport AUD and most likely the registered cases will not be representative of individuals suffering from AUD. In this perspective, the present study provides an important addition to the existing studies by including a broader range of individuals with AUD and not just individuals admitted to a psychiatric hospital department. Compared with previous studies, the present study may also provide a better sample for studying comorbidity among individuals with AUD because it was based on individuals who specifically sought treatment for this disorder. In contrast, most of the previous studies were based on individuals receiving general inpatient psychiatric treatment (Flensborg‐Madsen et al., 2009; Gale et al., 2010; Mortensen et al., 2005; Tomasson & Vaglum, 1995), and therefore, these studies may to a large extent reflect how psychiatric hospital diagnoses co‐occur among individuals in a psychiatric hospital setting rather than the pattern of psychiatric comorbidity in a population of individuals with AUD.

*Other non‐psychotic disorders* were also prevalent in the present study (32.0%), but none of the previous studies included a combined category of other non‐psychotic disorders. However, several of the previous studies found that neuroses and anxiety disorders were prevalent among individuals receiving AUD treatment with prevalence estimates up to 52.3% (Burns et al., 2005; Gale et al., 2010; Mellentin et al., 2015, 2018; Nordholm & Nielsen, 2007; Schneider et al., 2001; Smith & Book, 2010; Tomasson & Vaglum, 1995; Yoshimi et al., 2016). In contrast, another study reported that the prevalence of anxiety disorders was 6.6% among individuals receiving AUD treatment (Flensborg‐Madsen et al., 2009), which was similar to the prevalence of 8.6% observed in the present study. Hence, the prevalence of neuroses and anxiety disorders varied markedly between the previous studies and the present study, but also within the previous studies, which may be a result of several factors. First, the previous studies were based on populations receiving different types of AUD treatment. Thus, some of the studies included individuals receiving inpatient AUD treatment (Gale et al., 2010; Kushner et al., 2005; Schneider et al., 2001; Tomasson & Vaglum, 1995), other included individuals receiving outpatient AUD treatment (Burns et al., 2005; Mellentin et al., 2015, 2018; Nordholm & Nielsen, 2007; Smith & Book, 2010; Yoshimi et al., 2016), and one included individuals receiving both inpatient and outpatient AUD treatment (Flensborg‐Madsen et al., 2009). Second, the studies were based on populations from different countries including Denmark (Flensborg‐Madsen et al., 2009; Mellentin et al., 2015, 2018; Nordholm & Nielsen, 2007), Iceland (Tomasson & Vaglum, 1995), Sweden (Gale et al., 2010), Germany (Schneider et al., 2001), Australia (Burns et al., 2005), Brazil (Yoshimi et al., 2016), and the United States (Kushner et al., 2005; Smith & Book, 2010), possibly reflecting different traditions of registration and treatment of psychiatric disorders. Third, the studies used different diagnostic tools to assess psychiatric comorbidity including the International Classification of Diseases (ICD‐8 and ICD‐10) (Flensborg‐Madsen et al., 2009; Gale et al., 2010; Mellentin et al., 2015, 2018; Nordholm & Nielsen, 2007; Schneider et al., 2001), Diagnostic and Statistical Manual of Mental Disorders (DSM, version III and IV) (Burns et al., 2005; Kushner et al., 2005; Schneider et al., 2001; Smith & Book, 2010; Tomasson & Vaglum, 1995), and The Social Phobia Inventory (Yoshimi et al., 2016). Finally, some studies reported lifetime prevalence estimates of neuroses and anxiety disorders (Flensborg‐Madsen et al., 2009; Gale et al., 2010; Nordholm & Nielsen, 2007; Tomasson & Vaglum, 1995), while one reported the 12‐month prevalence (Burns et al., 2005), and other reported the current prevalence (Kushner et al., 2005; Mellentin et al., 2015, 2018; Schneider et al., 2001; Smith & Book, 2010; Yoshimi et al., 2016).

In addition to other nonpsychotic disorders, *personality disorders* were prevalent in the present study with a prevalence estimate of 25.3%. Personality disorders were also prevalent in the previous studies of individuals receiving AUD treatment, but like the results on neuroses and anxiety disorders, the prevalence estimates of personality disorders varied notably, ranging from 24.0% to 34.0% (Flensborg‐Madsen et al., 2009; Mortensen et al., 2005; Nordholm & Nielsen, 2007). Similar mechanisms as those described for neuroses and anxiety disorders may explain the large variation in the prevalence estimates for personality disorders.

Finally, the prevalence of *mental retardation* (1.4%) and *development disorders* (1.2%) was low in the present study. However, apart from one of the previous studies reporting a relatively high prevalence of cognitive impairment (16.0%) (Tomasson & Vaglum, 1995), none of the existing studies included mental retardation or development disorders as separate categories (Flensborg‐Madsen et al., 2009; Gale et al., 2010; Mortensen et al., 2005; Tomasson & Vaglum, 1995). This may reflect a general limited use of these diagnoses, which was also found in a Danish nationwide study of psychiatric hospital diagnoses, observing low occurrences of both mental retardation and developmental disorders (Pedersen et al., 2014). Especially, the low prevalence of developmental disorders is surprising as this disease category included attention deficit hyperactivity disorders (ADHD) that are highly comorbid with AUD (Katzman et al., 2017). However, the use of ADHD diagnoses has increased markedly over the last decade (Davidovitch et al., 2017), while the present study included older birth cohorts, possibly explaining the incongruence.

### 4.2 Comparisons with the control population

The present study observed that the prevalence of lifetime psychiatric hospital diagnoses was almost four times larger among men registered in an outpatient alcohol clinic compared with the control population. This is both in line with results from a treatment sample (Schuckit et al., 1997) and a population sample (Farrell et al., 2003) observing that the prevalence of psychiatric comorbidity was more than double among individuals with AUD compared with individuals without AUD. In addition, population surveys have found that individuals fulfilling the diagnostic criteria for AUD had significantly larger ORs of lifetime substance use disorders, mood disorders, anxiety disorders, and personality disorders with ORs ranging from 1.4 to 15.8 compared with individuals not fulfilling the diagnostic criteria for AUD (Hasin et al., 2007; Ross, 1995). These results are also congruent with the results from the present study, finding significantly larger ORs of lifetime substance use disorders, mood disorders, anxiety disorders, and personality disorders among individuals registered in an outpatient alcohol clinic compared with the matched control population.

### 4.3 Temporal order of registration with an AUD diagnosis and another psychiatric hospital diagnosis

*The AUD first subpopulation* included 1,035 men and may reflect situations where AUD was the primary factor for seeking treatment. Thus, this subpopulation may include cases where AUD affected the development of another psychiatric disorder directly or indirectly reflected as effects of alcohol‐related social conflicts and stress. However, it is also possible that this subpopulation includes men with prior co‐occurring disorders that did not require hospital treatment (i.e., was treated in primary care). Finally, it is also likely that this subpopulation contains men who developed AUD as a consequence of using alcohol to cope with symptoms of undiagnosed prior psychiatric disorders, possibly reflected in the significantly higher prevalence of mood disorders in the AUD first subpopulation compared with the other two subpopulations.

Among men with a lifetime psychiatric hospital diagnosis, the majority was registered with another psychiatric hospital diagnosis prior to registration with an AUD diagnosis (42.8%). This indicates that among men with psychiatric comorbidity, AUD is often diagnosed after another psychiatric hospital diagnosis. In fact, AUD was only diagnosed without registration of co‐occurring psychiatric disorders in 11.9% of the first‐time admission to a psychiatric hospital department. Hence, *the other diagnosis first subpopulation* probably reflects cases where other psychiatric disorders than AUD were the primary reason for hospitalization. Specifically, the significantly higher prevalence of other psychotic disorders than schizophrenia observed in this subpopulation compared with the AUD first subpopulation may suggest that severe psychiatric disorders lead to initial contact with a psychiatric hospital department and that AUD is diagnosed later in these cases. However, among individuals with AUD, relatively low treatment‐seeking rates have been observed (Chapman et al., 2015; Cohen et al., 2007; Dawson et al., 2005; Hasin et al., 2007) and the individuals who eventually seek treatment for the disorder often wait several years to do so (Cohen et al., 2007; Hasin et al., 2007; Hingson et al., 2006; Keyes et al., 2010; ten Have et al., 2013). Hence, the other diagnosis first subpopulation may also include cases with prior undiagnosed AUD. Nonetheless, the high prevalence of AUD hospital diagnoses in this subpopulation suggests that eventually AUD is diagnosed, even in cases where the first admission is unrelated to AUD.

*The simultaneous diagnosis subpopulation* had a significantly higher prevalence of most lifetime psychiatric hospital diagnoses compared with the other two subpopulations. This was expected as this subpopulation was defined as having at least two lifetime psychiatric hospital diagnoses, but it also indicates that AUD and other psychiatric disorders often co‐occur and are diagnosed at the same age. One possible explanation of this co‐occurrence is that there exists a certain bidirectionality between AUD and other co‐occurring disorders. This was, for example, demonstrated in a Danish register‐based study, observing that AUD increased the risk of other psychiatric disorders, while the opposite also was true as the presence of another psychiatric disorder increased the risk of AUD (Flensborg‐Madsen et al., 2009). Another possible explanation is that there exist common causes including mental characteristics such as the personality trait neuroticism (Kotov et al., 2010; Lönnqvist et al., 2009) and intelligence (Gale et al., 2010, 2013; Rommelse et al., 2016) that can impact the risk of both AUD diagnoses and other psychiatric hospital diagnoses.

### 4.4 Strengths and limitations

The current study carries several strengths. First, the analyses were based on a large population of 8,412 men, which increased the statistical power to detect prevalence differences and reduced random error. In addition, we included a matched control population of 8,412 men never registered in an outpatient alcohol clinic, making it possible to evaluate psychiatric comorbidity among men registered in an outpatient alcohol clinic. Third, we included, virtually, the full spectrum of treated lifetime psychiatric hospital diagnoses in a Danish psychiatric hospital setting, using both ICD‐8 and ICD‐10. This allowed a thorough assessment of psychiatric comorbidity and the temporal order of registration with an AUD diagnosis and registration with another psychiatric hospital diagnosis, although the use of both ICD‐8 and ICD‐10 may have led to the identification of different phenotypes. Next, information on lifetime psychiatric hospital diagnoses can be considered of high quality as it was retrieved from registers with a systematic data collection and high coverage (Lynge et al., 2011; Munk‐Jorgensen & Mortensen, 1997). However, psychiatric disorders treated in a primary care setting were not included in the present study, and the prevalence of lifetime psychiatric hospital diagnoses may be underreported or lacking for some men if they sought psychiatric treatment before 1969 as we only have data from the national Danish psychiatric registers from this year and onwards. In addition, the diagnostic procedure may also have led to underreporting of psychiatric comorbidity. This may both imply that some men who appeared not to have a lifetime psychiatric hospital diagnosis did, in fact, have one and that some men who were registered in an outpatient alcohol clinic prior to registration with a psychiatric hospital diagnosis had the opposite temporal order. Finally, our study was based on a sample of men registered in an outpatient alcohol clinic and it is not unlikely that the probability of being registered with a lifetime psychiatric hospital diagnosis is different in these men compared with the general population or individuals receiving other types of AUD treatment. Hence, the results cannot be generalized to all men with AUD.

In conclusion, 59.4% of the men registered in an outpatient alcohol clinic had a lifetime AUD hospital diagnosis, indicating that studies based solely on psychiatric hospital diagnoses will underreport AUD and only include a selected group of individuals with psychiatric comorbidity.

The odds ratio of having a lifetime psychiatric hospital diagnosis was more than nine times larger among men registered in an outpatient alcohol clinic compared with the control population. However, among men with a lifetime psychiatric hospital diagnosis, 11.9% was registered with an AUD hospital diagnosis prior to registration with another psychiatric hospital diagnosis, indicating that AUD rarely is diagnosed without co‐occurring psychiatric disorders at first‐time admissions to a psychiatric hospital department.

## CONFLICTS OF INTEREST

None.

## AUTHOR CONTRIBUTIONS

All authors have made substantial contributions to the design and conception of the study. LANC analyzed the data and drafted the manuscript. ELM, MO, HJS, UB, and TFM critically revised the manuscript for important intellectual content. All authors approved the final version of the manuscript and agreed to be accountable for all parts of the work.

### Peer Review

The peer review history for this article is available at https://publons.com/publon/10.1002/brb3.2004.

## Data Availability

Research data are not shared.
